# Applications of natural language processing in ophthalmology: present and future

**DOI:** 10.3389/fmed.2022.906554

**Published:** 2022-08-08

**Authors:** Jimmy S. Chen, Sally L. Baxter

**Affiliations:** ^1^Division of Ophthalmology Informatics and Data Science, Viterbi Family Department of Ophthalmology and Shiley Eye Institute, University of California San Diego, La Jolla, CA, United States; ^2^Health Department of Biomedical Informatics, University of California San Diego, La Jolla, CA, United States

**Keywords:** natural language processing, ophthalmology, artificial intelligence, machine learning, big data, informatics, data science

## Abstract

Advances in technology, including novel ophthalmic imaging devices and adoption of the electronic health record (EHR), have resulted in significantly increased data available for both clinical use and research in ophthalmology. While artificial intelligence (AI) algorithms have the potential to utilize these data to transform clinical care, current applications of AI in ophthalmology have focused mostly on image-based deep learning. Unstructured free-text in the EHR represents a tremendous amount of underutilized data in big data analyses and predictive AI. Natural language processing (NLP) is a type of AI involved in processing human language that can be used to develop automated algorithms using these vast quantities of available text data. The purpose of this review was to introduce ophthalmologists to NLP by (1) reviewing current applications of NLP in ophthalmology and (2) exploring potential applications of NLP. We reviewed current literature published in Pubmed and Google Scholar for articles related to NLP and ophthalmology, and used ancestor search to expand our references. Overall, we found 19 published studies of NLP in ophthalmology. The majority of these publications (16) focused on extracting specific text such as visual acuity from free-text notes for the purposes of quantitative analysis. Other applications included: domain embedding, predictive modeling, and topic modeling. Future ophthalmic applications of NLP may also focus on developing search engines for data within free-text notes, cleaning notes, automated question-answering, and translating ophthalmology notes for other specialties or for patients, especially with a growing interest in open notes. As medicine becomes more data-oriented, NLP offers increasing opportunities to augment our ability to harness free-text data and drive innovations in healthcare delivery and treatment of ophthalmic conditions.

## Introduction

Adoption of electronic health records (EHRs) and advances in ocular imaging technology have revolutionized healthcare delivery in ophthalmology and resulted in significantly increased data available for clinical care and research (1). Moreover, the breadth of available data has resulted in large, multimodal datasets that have enabled the revolution in “big data” analytics (1, 2). The American Academy of Ophthalmology (AAO) and National Institutes of Health (NIH) have supported this movement with the development of large, processed EHR-based datasets such as the Intelligent Research in Sight (IRIS) Registry (3, 4) and the *All of Us* research program (5). Research efforts using these datasets have largely focused on retrospective association analysis and trends in care (6–14). Large datasets have also been used to develop predictive artificial intelligence (AI) models. The majority of these applications within ophthalmology have focused on image-based AI including diagnosis of diabetic retinopathy (15, 16), age-related macular degeneration (17, 18), retinopathy of prematurity (19, 20), and glaucoma (21–23), among others. Though structured datasets (such as extracted tabular data from EHRs) and large image datasets have been studied extensively in ophthalmic big data applications, far fewer AI studies in ophthalmology have utilized unstructured, or free-text, data such as EHR clinical notes from office visits (24–27). Because clinical notes represent the majority of provider documentation regarding each office visit, there remains a large amount of untapped free-text data (up to 80% of data in the EHR) that may be useful in predictive AI or analytics (28).

Natural language processing (NLP) is a subfield of AI focused on extracting and processing text data, including written and spoken words. While NLP as a linguistic concept originated in the early 1900s, it did not gain widespread interest until the last few decades with the proliferation of computer-based and AI algorithms. Within medicine, NLP has primarily been used for information retrieval (IR, otherwise known as search) (29, 30), text extraction for analytic studies, and AI algorithm development, though recent studies have focused on more complex tasks such as question-answering and summarization. Furthermore, there is a dearth of studies exploring the use of NLP in ophthalmology. Because ophthalmology is a high-volume medical and surgical subspecialty, there are significant opportunities to take advantage of the wealth of available data to develop text-based technologies with the potential to improve patient care and enhance future research.

The purpose of this study was to introduce ophthalmologists and researchers to natural language processing by (1) reviewing current ophthalmic applications of NLP, and (2) discussing future opportunities for NLP in ophthalmology.

### Natural language processing

**Figure 1:**
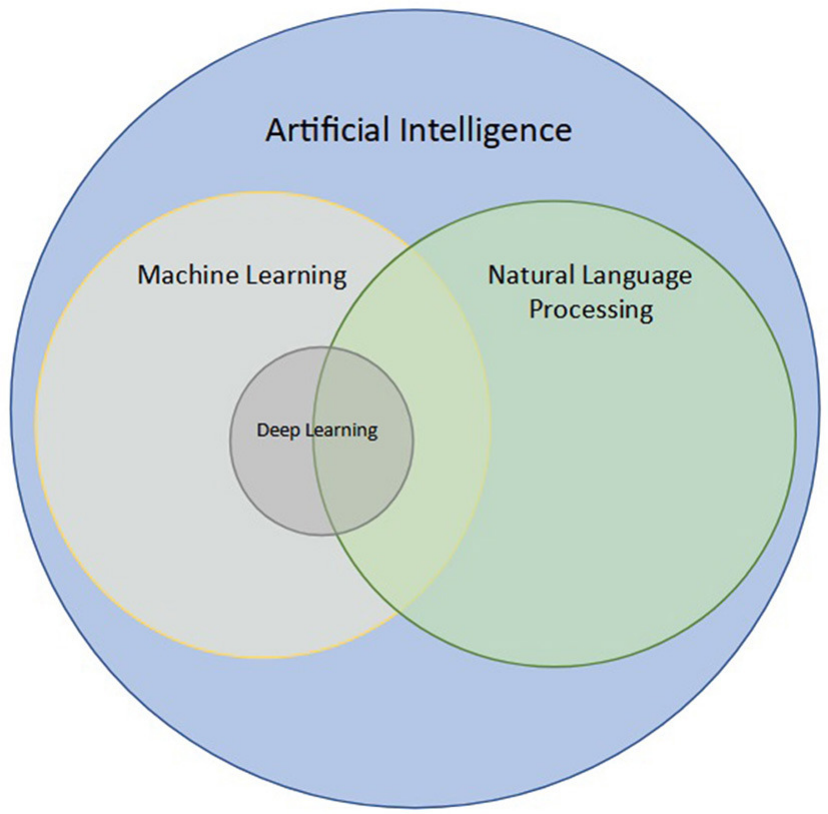
Intersection of natural language processing (NLP) with artificial intelligence (AI), machine learning (ML), and deep learning (DL). NLP is a branch of AI concerned with processing and analyzing text data. ML is a subfield of AI aimed at modeling data, and DL is a subfield of ML that uses neural networks to analyze large datasets. NLP techniques may utilize ML and DL when used for classification of words, sentences, or even paragraphs.

In simple terms, the goal of NLP is to learn meaning from a set of words. However, the distinction between NLP, AI, and machine learning (ML) is often unclear. Broadly, AI is the branch of computer science that deals with teaching computers to perform tasks ordinarily performed by humans (31, 32). ML is a branch of AI that deals with developing models for automated prediction of a given task (33–35). Within ML, modeling can be performed using neural networks, which have the ability to learn from large amounts of data without explicitly defined features, an area known as deep learning (DL) (33, 36). While several NLP techniques that do not utilize modeling such as ML or DL exist, some NLP can be used to perform modeling with ML or DL, using free-text (raw or processed) as input rather than images or pre-defined features (i.e., tabular data, or data in tables) (27, 37). This intersection of ML, DL, and NLP is shown in Figure 1.

Before the advent of computational NLP techniques, search methods originally focused on simple keyword extraction. At the most basic level, this was analogous to the “find” function in a word processor, where a body of text was searched for all instances of a specific word or phrase, often described as a regular expression, or regex, in computer science. Relatively more advanced search could be performed using rule-based search, or conditional searching, such as extracting a word if it was in the sentence with another word. In fact, this concept of search, otherwise known as information retrieval (IR), is an important cornerstone of NLP (38, 39). However, the primitive methods described above are limited by the need for manual search input and a prior understanding of the text involved.

More sophisticated methods of text extraction require understanding the context of each input word in a body of text. This is most commonly done by labeling specific words as entities, which can include person, location, etc., a technique known as named entity recognition (NER). This is often done in conjunction with relation extraction (RE), which focuses on how phrases relate to others (i.e., patient underwent “4 cycles” of chemotherapy, where 4 cycles defines duration). However, these techniques often require pre-processing words within a given text to their simplest form. This usually begins with tokenization, or splitting a body of text into its individual words, and transforming all words to lowercase. Further text-preprocessing includes stemming (reducing a word down to its base form often with misspellings; i.e., “changes,” “changing” becomes “change”), lemmatization (simplifying a word down to its simplest form - i.e., “changes” to “change”, or “different” to “differ”), as well as stop-word removal (i.e., removing common words to simplify data analysis; most commonly articles like “a” and “the” are removed). Once a text has been pre-processed, NER techniques can be used to perform tasks such as de-identification, automated search, or annotating specific words (i.e., medications in progress notes). De-identification in particular has been a recent focus of research in NLP (40–42), and typically involves using text negation, or censoring out specific words of interest such as patient health information (PHI). NER can also be augmented by tagging each word's part of speech (43). In medicine, existing NLP models for NER such as MedEx (44) and MedLEE (45), which identify medications and diagnostic entities for billing, respectively, have been previously developed without ML. Off-the-shelf NER models for medical information extraction have also been provided by Amazon Comprehend Medical and require no prior programming knowledge, which has implications for increasing the accessibility for NLP engagement to the general public.

**Figure 2:**
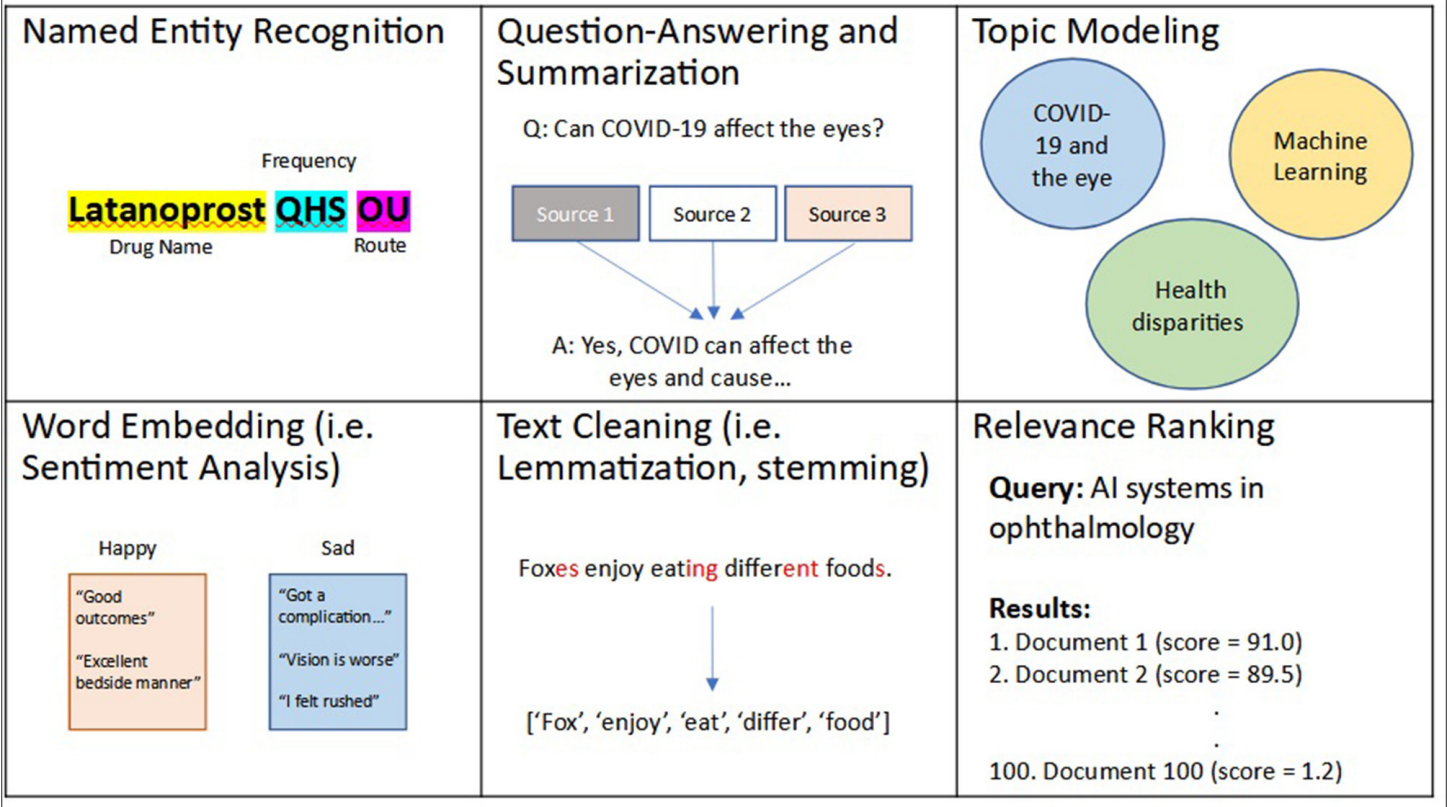
Examples of natural language processing (NLP) techniques and applications. Natural language processing, or NLP, is an area of artificial intelligence (AI) that deals with processing and analyzing textual data. Several NLP techniques include: relevance ranking, named entity recognition (NER), text cleaning, word embedding, which has applications in question-answering, summarization, topic modeling, among several other use cases.

Recently, NLP techniques have utilized ML and DL to perform more intelligent and complex textual tasks. For example, several state-of-the-art algorithms have utilized ML and DL to create more robust and efficient NER algorithms, including open-source software libraries such as spaCy (46). However, these algorithms are unable to recognize similarities and differences between words (i.e., “happy” is similar to “joy” but different from “sad”). A simple method to capture word similarity is a bag-of-words approach, commonly implemented as term frequency-inverse document frequency (TF-IDF). In this approach, a numerical value is essentially assigned to each unique word, though this approach is limited by its ability to recognize synonyms and more complex relationships between words. To address this gap, word embedding was developed. Simply put, word embeddings, such as word2Vec (47), are developed as a result of DL algorithms that learn to assign a numerical distance to 2 words, and are trained to do so on many combinations of words based on the corpora of text used for training. These algorithms have previously been fine-tuned on several datasets including Google News and a combination of EHR and biomedical corpora (48). Current state-of-the-art word embedding algorithms have utilized more complex neural networks, known as transformers, to automate complex analysis of contexts between words. These algorithms, the most common of which is known as Bidirectional Encoder Representations from Transformers (BERT), introduce the idea of attention, of the ability to focus on specific words and their complex relationships, and have transformed our ability to perform text processing (49). BERT models have also been trained on biomedical text and include: clinicalBERT trained on EHR notes (50) and bioBERT trained on biomedical publications (51). Common applications of word embedding algorithms include tasks such as: question-answering, summarization (52–57), topic modeling (58–61), creating recommendation systems (62–64), chatbots (65–68), voice recognition (i.e., speech-to-text) (69, 70), text translation (71, 72), ranking texts for relevance based on a search query (73–75), and sentiment (emotion) analysis (76–78). A summary of these aforementioned described techniques and applications is shown in Figure 2.

## Methods

**Figure 3:**
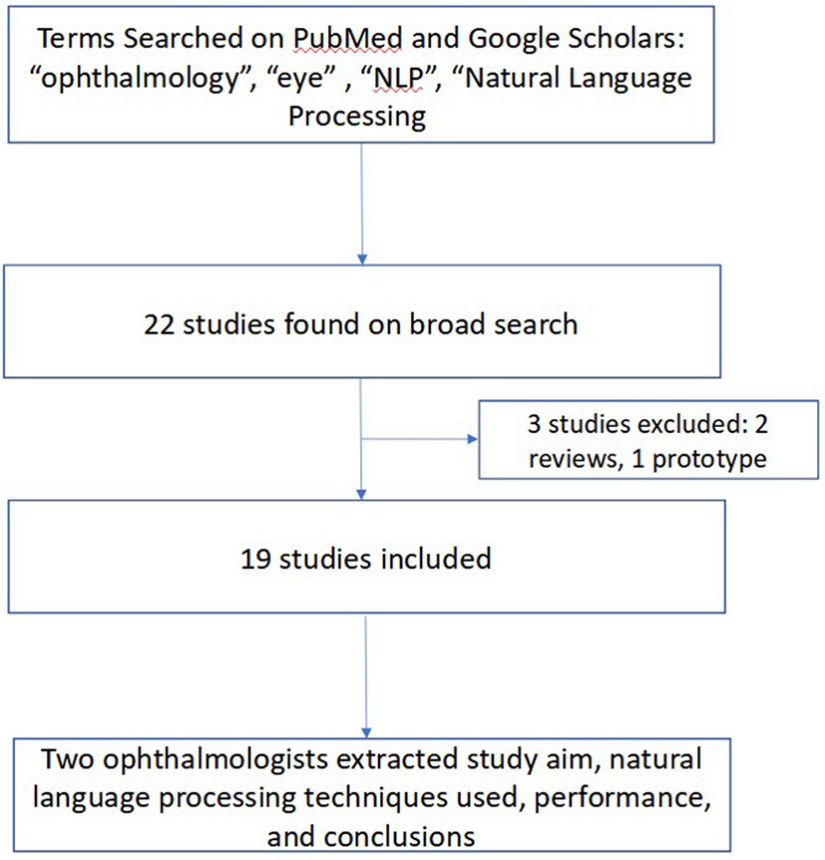
Methodology for Review of Ophthalmic Studies Utilizing Natural Language Processing (NLP). We searched PubMed and Google Scholars, augmented by ancestor search for studies related to use of NLP in ophthalmology applications.

To conduct this narrative review, a keyword-based and medical subject headings (MeSH)-based search of Pubmed and Google Scholars was performed in March 2022 using a combination of the following terms: “ophthalmology”, “optometry”, “eye”, “natural language processing”, and “NLP” to identify studies that used NLP in an ophthalmic context. These terms were combined in several different combinations and permutations in both search engines to yield an initial yield of 22 studies. Studies were included if they described original research using NLP in ophthalmology. All studies designed as a literature review or prototype description were excluded. Ancestor search was performed on included studies to further broaden our references. Both authors (JSC and SLB) manually reviewed each study's title, abstract, and manuscript text to validate the relevance of the studies to both ophthalmology and NLP. Data extracted from each study included: the authors and year of publication, study aim, NLP techniques used, performance, and study conclusions. Disagreements were resolved by discussion. This methodology is summarized in Figure 3.

## Results

### The present: Current ophthalmic studies using NLP

Overall, 19 studies using NLP in ophthalmology were identified in the literature. These studies were published between 2000 and 2022, of which the majority (*n* = 11, 58%) were published within the last 3 years (2019–2022). Initial NLP studies did not use ML and focused mostly on algorithmic text extraction of relevant text from clinical notes using rule-based search and keyword extraction for parameters such as visual acuity (VA) (79–81), demographic data (i.e., age, sex) as well as clinical data (i.e., intraocular pressure, visual acuity) related to glaucoma (82) and cataract identification (83). Subsequent studies focused on using similar algorithmic rule-based search retrieving text relevant to the diagnosis and identification of several diseases such as herpes zoster ophthalmicus (84), pseudoexfoliation syndrome (85), microbial keratitis (25), and fungal endophthalmitis (24). While most published work has focused on extracting information from clinical visit notes (24, 84, 86), Stein et al. extracted a combination of unstructured data, problem lists, clinical notes, and billing code documentation for multi-modal extraction of pseudoexfoliation syndrome (85). Other use cases for text extraction using search included identifying antibiotics used for and post-operative complications of cataract surgery (87), extracting eye laterality and medications of patients who underwent cataract surgery (88), as well as for triaging ophthalmology referrals (89).

In the last 3 years, more recent studies have begun using ML for more sophisticated applications of NLP. For example, Wang et al. created the first word embeddings specific to ophthalmology using ophthalmology publications and EHR notes and found that DL models trained on ophthalmology-specific word embeddings outperformed those trained on previous word embeddings trained on general vocabulary for predicting prognosis of low-vision (90). These embeddings were also later used in combination with structural, tabular data from the EHR to refine models predicting low-vision prognosis (91). This idea of combining structured and unstructured data from the EHR was also applied to predicting glaucoma progression using similar methods described earlier (92). Additionally, Lin et al. recently applied an existing DL framework for NER to accurately extract entities relevant to ophthalmic medications (F1 score = 0.95) for glaucoma patients and simulated successful medication reconciliation as an application of this NLP model (93). The F score has become an increasingly popular metric to evaluate model performance in NLP, and measures both the precision (positive predictive value) and recall (sensitivity). These F scores can be weighted (with weights appended to the score name - i.e., F1, F2 scores) to increase the importance of maximizing either precision or recall. Other recent studies utilizing ML/DL with NLP included topic modeling to define groups of topics pertaining to ophthalmology publications during the COVID-19 pandemic (94), as well as sentiment analysis of user emotions from an ophthalmology forum (95). Topic modeling uses unsupervised learning, or machine learning without explicitly labeled data, to cluster documents by topic. In work performed by Hallak et al., the authors used a statistical model called Latent Dirichlet Allocation (LDA) to identify ocular manifestations of COVID-19, viral transmission, patient care, and practice management during the COVID-19 pandemic as relevant topics in ophthalmology over 2020–2021 (94). Additionally, in work by Nguyen et al., a cloud-based NLP program called Watson was utilized to associate emotions with extracted keywords from ophthalmology forums and demonstrated that NLP can be used to understand patient perspectives on care. A summary of these studies is shown in Table 1.

**Table 1:** Summary of Current Studies using NLP in Ophthalmology.

**References**	**Year**	**Aim**	**NLP techniques**	**Study outcomes**	**Conclusions**
Barrows et al. (82)	2000	Automated extraction of demographic and clinical parameters relevant to glaucoma from EHR notes	Text data extraction (Rule-based search, MedLEE)	All parameters were extracted with a >90% accuracy	Rule-based search performed similarly to NLP methods in terms of extracting text data from the EHR
Smith et al. (79)	2008	Extract visual acuity and diabetic retinopathy stage for analysis of quality of life	Text data extraction (Rule-based search)	No specific performance metrics for accuracy of text extraction was reported	NLP can identify visual acuity of patients with diabetic retinopathy for use in secondary regression analysis to predict quality of life with vision loss
Peissig et al. (83)	2012	Automated identification of cataracts from free-text and image-based EHR scans of text	Text data extraction (Rule-based search, MedLEE)	Positive predictive value >95%	Multi-modal strategies are highly accurate in identifying cataracts and generalizable across institutions
Mbagwu et al. (80)	2016	Extract Visual Acuity from defined structured fields and clinical notes in the EHR	Text data extraction (Rule-based search)	99% agreement between clinician and algorithm	Best corrected visual acuity can be automatically extracted from clinical notes in the EHR with high accuracy
Liu et al. (87)	2017	Extract antibiotics and intraoperative complications from cataract surgery operative notes	Text data extraction (Rule-based search)	Positive and negative predictive values for identification of antibiotic injection were >99%. For operative complications, extraction accuracy was >94%	NLP can extract data from operative notes with high accuracy
Baughman et al. (141)	2017	Automated visual acuity extraction from free-text clinical notes	Text data extraction (Rule-based search)	Manually reviewed and automated extracted visual acuities had a 95% concordance, K = 0.94	Automated visual acuity extraction from EHR free text notes is highly accurate
Gaskin et al. (81)	2017	Extract features from text notes using NLP for regression analysis	Text data extraction (Text negation and extraction using a predefined ontology)	No specific performance metrics for accuracy of text extraction was reported	Automated extraction of demographic factors, systemic disease, and drug information can be used to create high-performing models of cataract surgery complications
Zheng et al. (84)	2018	Extract diagnosis of herpes zoster ophthalmicus from EHR notes	Text data extraction (negating, lemmatization), part-of-speech tagging, indexing, tokenization, Classification	Sensitivity = 95.6%, specificity = 99.3%	NLP algorithms can classify herpes zoster ophthalmicus vs. not based on progress note data
Stein et al. (85)	2019	Identify exfoliation syndrome in free-text EHR notes	Text data extraction (Rule-based search)	Positive predictive value = 95% and Negative predictive value = 100%	Automated extraction of exfoliation syndrome appears to be more accurate than conventional assessment of billing codes
Maganti et al. (25)	2019	Extract microbial keratitis morphology measurements from free-text in the examination notes	Text data extraction (Rule-based search)	Microbial keratitis measurements were extracted with a sensitivity of 75–96% and specificity of 91–96%	Metrics of microbial keratitis can be automatically extracted from exam data in EHR notes
Tan et al. (89)	2019	Use NLP on free-text referrals to develop ML models for triaging	Text data extraction (Text negation, stripping), Classification	No specific performance metrics for accuracy of text extraction were reported	NLP can facilitate the training of ML models for triage
Baxter et al. (24)	2020	Identify fungal ocular involvement from EHR notes	Text data extraction (Rule-based search)	Rule-based search yield 683/26,830 notes with possible fungal ocular involvement. Manual review found 0% fungal ocular involvement.	NLP can expedite review of notes for fungal ocular disease
Wang et al. (88)	2020	Extract concepts related to vision outcomes from free-text notes and medication orders from the EHR	Text data extraction (Rule-based search, MedEx)	Rule-based laterality classifier: 100% accuracy. Implant usage: 99–100% accuracy. Glaucoma medications: 90.7% inter-annotator agreement, 85% accuracy for medications extracted by MedEx	NLP can be used to accurately extract laterality, medications, and implant model usage from cataract and glaucoma surgeries
Hallak et al. (94)	2020	Identify focuses of research in ophthalmology and AI related to the COVID-19 pandemic	Topic modeling	>200 manuscripts: 57.8% focused on patient care and practice management, 19.4% on transmission, 17.2% on ocular manifestations, 5.6% on treatment/diagnosis	NLP can identify recent focuses of ophthalmic research during the COVID-19 pandemic including ocular manifestations and applications of AI
Wang et al. (90)	2021	Create ophthalmology-specific word embeddings using published literature and EHR notes	Word embedding, Classification	PubMed and EHR word-embeddings resulting in similar AUROCs (~0.83) and outperformed previous non-ophthalmic word embeddings in predicting low vision prognosis	Ophthalmology-specific word embeddings can be used to increase prediction accuracy on prognosis of low-vision patients from EHR notes
Nguyen et al. (95)	2021	Utilize clinical data from social media and forums to understand patient attitudes toward care	Text data extraction (Rule-based search and text stripping into keywords), Sentiment analysis	Complications, body parts, and undiagnosed symptoms were associated with sadness. Joy was slightly more likely to be expressed after doctor response.	Sentiment analysis can be used to better understand patient perspectives and promote patient-centered care
Gui et al. (91)	2022	Use structured and extracted unstructured data to create models to predict low vision prognosis	Text data extraction (stripping), named entity recognition, word embeddings, classification	Several NLP techniques performed comparably well for predicting low vision prognosis (F1 0.63–0.7)	Free text progress notes can be used to accurately predict low vision prognosis using various NLP techniques
Wang et al. (92)	2022	Use extracted free-text data from notes and structured data to predict glaucoma progression to surgery using deep learning	Text data extraction (stripping), word embeddings, classification	Convolutional neural networks trained on structured + unstructured inputs outperformed models trained on structural features alone (F1 0.42 vs. 0.34) and both outperformed a glaucoma clinician (F1 0.30)	Structured and unstructured data from the EHR can predict glaucoma progression with accuracy
Lin et al. (93)	2022	Extract medication data from free-text progress notes	Named entity recognition	Overall F1 score = 0.95 for all medication entities (drug name, route, frequency, etc.)	Medication information can be accurately extracted from free-text data

Overall, 19 studies using NLP in an ophthalmic context were identified from the literature. The majority of these studies primarily used rule-based search as their use case for NLP, though more recent studies have begun using more sophisticated techniques such as word embedding and named entity recognition. These studies spanned publication from 2000 to 2022, with the majority written after 2019.

## Discussion

### The future: Opportunities for NLP in ophthalmology

Ophthalmology is a surgical subspecialty that could significantly benefit from applications of NLP, though there is a relative scarcity of published studies compared to those exploring NLP in other areas of medicine. Future avenues of exploration within ophthalmology include: (1) more complex use cases for text extraction, (2) translating notes both in terms of language, as well as (3) applications to assist with patient interaction.

While most studies within ophthalmology have focused on searching for specific keywords or entities, text extraction can be more broadly used for other use cases. For example, cohort selection, particularly for rare diseases, is a necessary prerequisite for clinical trial recruitment, and has been facilitated in the past by NLP algorithms reviewing EHR notes. In the 2018 National NLP Clinical Challenge for cohort identification, the highest performing model achieved an F-score of 0.9 for identifying cohorts using various criteria (96). Within inherited retinal diseases, cohort identification has been recognized internationally as an important goal in research with rapid advances in gene therapy; (97) however, a previously published current cohort identification study within this space focused on simple keyword search without use of more sophisticated NLP techniques (98). Additionally, drug repurposing has long been of interest to the medical community (99–101), and has been employed in mouse models for inherited retinal diseases (102, 103) as well as hypothesis testing for ocular protection against COVID-19 (104). While ophthalmology stands to greatly benefit from drug repurposing (105), the majority of applications using NLP have been published exploring novel drug use in cancer (106, 107) and COVID-19 (108, 109). However, within ophthalmology (110), one study by Brilliant et al. retrospectively demonstrated that L-DOPA could have protective effects against development AMD. Although drugs were quickly repurposed owing to the urgency of the COVID-19 pandemic, there remains a need for further exploration and prospective validation of potential drug candidates for repurposing both within ophthalmology and other specialties. As our techniques and capabilities for big data collection and analytics rapidly advances, more research is needed in both cohort identification and drug repurposing using NLP techniques and may have important implications in accelerating new innovations in ophthalmology.

NLP is also positioned to address challenges in interpreting documentation in the EHR by facilitating improved communication and understanding of clinician notes. NLP techniques centered around word embeddings have recently been utilized to develop question-answering (111–114), as well as summarizing large bodies of text such as clinical notes (52–54), and scientific publications (55, 115). With the advent of the Open Notes movement, a movement supporting transparent documentation among patients, families, and clinicians (116–118), and the 21st Century Cures Act of 2021 (119), which mandated patient accessibility to their clinical notes, there has been an increasing emphasis on patient involvement and advocacy in their own care. However, previous work in ophthalmology exploring clinician attitudes toward Open Notes revealed concerns that patients would have a difficult time understanding their records (120). In fact, the terminology used in ophthalmology notes have been anecdotally difficult to understand even among clinicians in other specialties, reflected by the creation of tools used by non-ophthalmologists to help “translate” ophthalmology notes by replacing common abbreviations used in ophthalmology (121). Summarization techniques may be useful to translate notes into patient-friendly language or even other languages (71) and may improve patient engagement in their healthcare, especially in underrepresented populations (72). However, in a systematic review by Mishra et al., the authors found that current work in NLP-based summarization focused largely on summarizing biomedical literature (97% of published work) as opposed to clinical data from the EHR (3% of published work), reflecting a need for work in NLP-based summarization in the clinical domain (56). Because ophthalmologists utilize specialized knowledge that is not commonly known to clinicians in other specialties, ophthalmology, as well as primary care specialties, stand to benefit significantly from tools that could summarize ophthalmic notes using NLP. Additionally, question-answering may have a role in extracting key data that would be most useful to facilitate management plans by primary care providers. While these technologies have numerous potential benefits, iterative testing and stakeholder participation will be needed to ensure that these NLP applications are useful and trustworthy by their users.

Patient interaction and patient-physician relationships remain the hallmark of medicine, but in areas with limited resources, NLP may be able to augment knowledge dissemination and assist clinician workflows. For example, chatbots using NLP have been previously developed to help patients with triaging concerns related to inflammatory bowel disease (65), recommending medical specialties based on symptoms (66), and other uses cases including depression symptom monitoring (67). Similar NLP-based chatbots may potentially be developed to assist with ophthalmic treatment monitoring and medication adherence as well as triaging ophthalmic symptoms for evaluation, particularly in areas where ophthalmology services may not be readily available. Additionally, NLP has been explored in the context of digital scribes, which have the potential to reduce physician burnout and increase patient satisfaction (122, 123). The burden of EHR-based documentation in ophthalmology has been well-described previously (124–127). A growing number of companies including Microsoft, Google, Amazon, IBM, Mozilla, DeepScribe, Suki, and Robin Healthcare have developed NLP-based scribes with speech recognition and smart medical assistants (122, 128–130). Because the majority of these NLP-based scribes are still in development, performance data to date is limited, though recent data from DeepScribe suggested that the model had an error rate of 18%, which is significantly lower than error rates from existing models by IBM and Mozilla (38–65%) (122). Development of these scribes have been complicated by technical challenges (i.e., audio quality, audio-to-text transcription) as well as conversational challenges (i.e., meaningful summarization, extracting topics from often fragmented conversations) (123). A recent study by Dusek et al. showed that scribe use in ophthalmology was associated with increased documentation efficiency (131). Automated scribes may potentially further increase documentation efficiency, and may be able to provide additional value if integrated with automated text extraction for providing relevant clinical information. Augmedix is another company attempting to integrate both remote scribing while providing data via Google Glass, though no NLP methods are currently used (132). Future research may focus on integrating NLP into these technologies to fully develop a “computer-based assistant” to assist with documentation, which may allow clinicians to focus on their relationship with the patient. Specifically in ophthalmology, counseling patients on preventing blindness, which remains the leading feared condition among American patients (133), requires significant investment in patient-physician relationships, and automated documentation could improve the quality of these relationships. These tools could also have additional value from a clinical workflow standpoint as ophthalmology is a high-volume specialty that requires processing of several data points and imaging modalities. While both chatbots and digital scribes are promising for optimizing the patient-physician relationship, significant refinement and iterative development of these systems is required before clinical deployment is feasible.

### Limitations of NLP

Though the future of NLP is exciting across all medical specialties including ophthalmology, there are important limitations that existing and future applications must address before use in the clinical setting. First, natural text is highly variable and error prone. Previous studies have shown that both dictated (7% error rate) (134) and written clinical notes (135) frequently contain errors such as documented actions that were not performed during the visit, findings from the visit that were not charted, as well as grammatical and typographical errors. Further, text often contains uses of words that can be used in different contexts, including colloquialisms, irony, sarcasm, and synonyms. These linguistic nuances often are difficult for NLP algorithms to distinguish. Second, word embeddings in NLP are often trained in specific domains [i.e., scientific publications (29), documents from web search (136)]. This importantly impacts how word embeddings interpret relationships between words, as different words may have different meanings in other contexts. Third, NLP models trained using DL or ML require huge datasets. These datasets are often difficult to acquire, and often need to be collected for a variety of settings (i.e., multi-institutional) to train a robust, generalizable model. Transformers, the current state-of-the-art DL method for NLP, require large datasets, with prior studies demonstrating worse performance on more limited datasets (137). Fourth, the majority of NLP applications have currently been developed in the English language (138). To promote equity in care and reduce healthcare disparities, more research is needed in developing NLP applications in non-English languages (71, 138–140), which has the potential to benefit populations with limited access to healthcare resources.

## Conclusion

NLP within ophthalmology is in its nascent stages of development and has already demonstrated potential in augmenting our ability to analyze free-text data from the EHR and improve predictive modeling with AI. As data from the EHR continues to grow, there remains significant opportunities to use NLP to improve our quality of research, “big data” analytics, and ultimately patient outcomes. However, there remain significant limitations of NLP that future work will need to address. More research and ongoing interdisciplinary collaborations will be needed to eventually translate NLP innovations into deployable solutions in the clinic.

## Author contributions

JC drafted the manuscript. SB provided overall supervision and guidance. All authors conceived the study, analyzed the data, interpreted the data, reviewed the manuscript, and revised for important intellectual content. All authors contributed to the article and approved the submitted version.

## Funding

This work was supported by NIH Grant DP5OD029610 (Bethesda, MD, USA) and an unrestricted departmental grant from Research to Prevent Blindness (New York, NY).

## Conflict of interest

The authors declare that the research was conducted in the absence of any commercial or financial relationships that could be construed as a potential conflict of interest.

## Publisher's note

All claims expressed in this article are solely those of the authors and do not necessarily represent those of their affiliated organizations, or those of the publisher, the editors and the reviewers. Any product that may be evaluated in this article, or claim that may be made by its manufacturer, is not guaranteed or endorsed by the publisher.
